# Genetic contributions to body mass index over adolescence and its associations with adult weight gain: a 25-year follow-up study of Finnish twins

**DOI:** 10.1038/s41366-024-01684-3

**Published:** 2024-11-20

**Authors:** Alvaro Obeso, Gabin Drouard, Aline Jelenkovic, Sari Aaltonen, Teemu Palviainen, Jessica E. Salvatore, Danielle M. Dick, Jaakko Kaprio, Karri Silventoinen

**Affiliations:** 1 Department of Genetics, Physical Anthropology and Animal Physiology, Faculty of Science and Technology, University of the Basque Country, Bilbao, Spain; 2 Institute for Molecular Medicine Finland, HiLIFE, University of Helsinki, Helsinki, Finland; 3 Department of Psychiatry, Robert Wood Johnson Medical School, Rutgers University, New Brunswick, NJ USA; 4 Helsinki Institute for Demography and Population Health, University of Helsinki, Helsinki, Finland

**Keywords:** Endocrinology, Epidemiology

## Abstract

**Introduction:**

High body mass index (BMI) in adolescence is a strong predictor of adult obesity. However, the nature of this association is unclear. We investigated how adolescent BMI is associated with adult weight change using longitudinal data from ages 11.5 to 37 years and examined the genetic factors behind these associations.

**Data and Methods:**

The study cohort consisted of 1400 Finnish twin individuals (40% males) with 494 complete twin pairs who reported their body mass index (BMI) at five ages: 11.5, 14, 17.5, 24, and 37 years. BMI trajectories (defined as BMI changes (i.e., slope) and BMI at baseline age (i.e., intercept)) were calculated in adulthood (from 17.5 to 37 years of age) using linear mixed-effects models. Polygenic Risk Scores of BMI (PRS_BMI_) and genetic twin models were utilised to analyse the role of genetic factors underlying BMI trajectories and their associations with BMI at 11.5 and 14 years of age.

**Results:**

Mean BMI increased in adulthood (4.06 kg/m^2^ in men and 3.39 kg/m^2^ in women). The BMI changes correlated with BMI at the baseline age of 17.5 years (i.e. intercept) (*r* = 0.24 in men and *r* = 0.35 in women) as well as with BMI in adolescence (11.5 and 14 years of age). Genetic factors contributed to the BMI changes during adulthood (correlation with PRS_BMI_ *r* = 0.25 in men and *r* = 0.27 in women; heritability estimates 0.63 and 0.64 respectively) as well as to their correlations with BMI at the baseline age (*r*_A_ = 0.5 in men and 0.54 in women) and BMI during adolescence (at 11.5 and 14 years of age) (*r*_A_ = 0.63–0.64).

**Conclusion:**

We found that genetic factors play a role in BMI change in adulthood, and part of this genetic component overlaps with the genetics of BMI in adolescence. Genetic predisposition to high BMI in adolescence is also related to adult weight gain.

## Introduction

Obesity is defined as an excessive accumulation of adipose tissue in the body leading to several health consequences, such as an increased risk of metabolic diseases, cardiovascular diseases, musculoskeletal diseases, and several cancers [1]. According to the World Health Organisation, the prevalence of obesity has almost tripled in the past 40 years, and approximately 3.12 billion people (nearly 40% of the global population) suffer from overweight or obesity [2]. Obesity rates are also on the rise in low- and middle-income countries [3]. Paediatric obesity is of interest as previous studies have shown it plays an important role as a risk factor for adult obesity [4] and predisposes individuals to increased health risks in adulthood [5, 6]. Furthermore, in adulthood, various physiological and psychological mechanisms act to prevent weight loss and promote weight gain [7]. Therefore, it is crucial to understand the mechanisms underpinning the continuity of body mass index (BMI) from childhood to adulthood.

Although obesity is a physiological consequence of excess caloric intake relative to expenditure, the underlying causes of this imbalance are complex. The development of obesity is influenced by a multitude of factors, including biological, developmental, environmental, behavioural, and genetic factors, as well as their interactions [8]. The role of genetic factors in the development of obesity is well documented [9, 10]. Previous twin studies involving children [11, 12] and adults [13] have reported high heritability estimates for BMI. Furthermore, the significance of genetics in obesity has been substantiated by genome-wide association (GWA) studies, which have identified a multitude of genetic variants influencing BMI variation in childhood [14] and adulthood [15] in cross-sectional analyses. Nevertheless, the genetics of weight gain remain poorly understood. Previous twin and family studies have demonstrated that genetic factors influence weight gain in adulthood, with heritability estimates ranging from 0.39 to 0.70 [9, 16, 17]. Moreover, a twin study based on data from the older cohort of the Finnish Twin Cohort Study demonstrated that there was a modest genetic correlation between baseline BMI and weight changes (*r*_A_ = −0.07 for men and *r*_A_ = 0.04 for women) [9]. However, all these studies were based on BMI measured in adults. Therefore, further investigation is required to ascertain the extent to which adolescent BMI is associated with BMI changes in adulthood, and whether these associations are due to shared genetic underpinnings.

The aim of this study is to examine the association between BMI during adolescence (ages 11.5 and 14) and BMI changes during adulthood (17.5 to 37 years of age) using longitudinal twin data from Finland. Additionally, the study aims to investigate whether the genetic predisposition to obesity is associated with BMI at different stages of life and its changes in adulthood. Two analytical approaches are employed in this study: genetic twin modelling and polygenic risk scores of BMI (PRS_BMI_). These are used to examine the shared genetic predisposition to obesity and BMI gain. The BMI at different ages was calculated from self-reported weight and height. BMI trajectories were obtained using linear mixed-effects models (LME), where BMI at baseline was defined as BMI at age 17.5, and weight change was examined from 17.5 to 37 years of age.

## Data and methods

The data were derived from the longitudinal FinnTwin12 study, which targeted all twins born in Finland between 1983 and 1987. A total of 5184 twin individuals participated in the baseline survey, with a response rate of 94%. The follow-up surveys were sent to the twin participants at the ages of 14, 17.5, 24, and 37, with response rates of 88%, 90%, 66%, and 43%, respectively [18, 19]. At each survey, participants were asked to report their current weight and height, which were then used to compute their BMI (kg/m^2^). The correlation between self-reported and measured BMI in young adulthood (wave 4) was found to be 0.97 showing good reliability of self-reported BMI [20, 21]. Zygosity was based on measured genotypes for the twins for whom we had a DNA sample. For the rest of the twins, zygosity was based on questions of physical similarity in the baseline questionnaire. This method was validated in this data set using 395 same-sex twin pairs, and in these twins, 97% of questionnaire assignments of zygosity were confirmed by a DNA test showing high reliability [22].

At survey wave 4, venous blood and saliva samples were provided by a subset of twins in the cohort (Rose et al. [18]). Even when BMI was not used to select these twins, their BMI was slightly lower compared to other participants (0.20–0.30 kg/m^2^) suggesting that there has been some self-selection related to BMI. Genotyping, quality control, and imputation were done using Illumina Human610-Quad v1.0 B, Human670-QuadCustom v1.0 A, Illumina HumanCoreExome (12 v1.0 A, 12 v1.1 A, 24 v1.0 A, 24 v1.1 A, 24 v1.2 A), and Affymetrix FinnGen Axiom arrays [23]. The PRS_BMI_ was derived using GWA summary statistics of BMI [15]. A total of 996,919 single nucleotide polymorphisms (SNPs) were employed in the PGS calculations, with a minor allele frequency of greater than 5% in European individuals. Subsequently, the data were processed using HapMap3 SNPs [15]. The number of individuals in the GWA studies was 692,578. The PRS_BMI_ was calculated for participants who had provided a DNA sample (*N* = 1478). In order to correct for population stratification (by incorporating the family identifier as random effects), we first regressed the PRS_BMI_ to the top ten genetic principal components [24]. Subsequently, the residuals were scaled to a mean of zero and a variance of one, and the PRSBMI was used unmodified in relevant analyses. The distribution of PRS values for the used sample (formed by those individuals with BMI measures for the last three waves; *N* = 1400) was similar when compared with the distribution of PRS values for the other participants of the FinnTwin 12 cohort (those that do not meet the criteria having BMI measures for the last three waves; *N* = 843). We also tested the genetic representativeness of our study participants by comparing the PRS density plots to two other Finnish twin cohorts (FinnTwin16 (FT16) and older Finnish Twin Cohort (FCT) Study using a two-sample Kolmogorov-Smirnov test [25, 26]. We did not find any differences in the PRS distribution of our study cohort as compared to FT16 study (*N* = 1312, *D* = 0.030767, *p*-value = 0.5276) or FCT (*N* = 11502, *D* = 0.015414, *p*-value = 0.916). This suggests that these cohorts genetically represent the general Finnish population.

Participants with BMI values for the last three waves (17.5, 24 and 37 years old) were selected to calculate weight gain trajectories using linear mixed-effects (LME) models. Longitudinal data analysis is well-suited to LME modelling, which incorporates both fixed and random effects to account for the correlation between longitudinal observations. For each individual, two values were derived from LME models: (i) an estimate of the rate of change in BMI (i.e., slope) and (ii) a baseline value (i.e., an intercept), collectively referred to as BMI trajectories. Analyses were performed using the R software (version 4.2.3) and the R packages lme4 (version 1.1-34), lmerTest (version 3.1-3), dplyr (version 1.1.4), modelsummary (version 1.4.3) and optimx (version 2023-10.21).

The contribution of BMI during adolescence to the longitudinal trajectory of BMI over adulthood was investigated by (i) assessing phenotypic correlations between the slope and intercept and (ii) between the slope and BMI at adolescence (ages 11.5 and 14). Further analyses involved examining (iii) the phenotypic correlations between the PRS_BMI_ and BMI at different ages and (iv) the changes in BMI over adulthood. The objective of these analyses was to assess whether genetic predispositions for higher BMI were associated with elevated BMI at specific ages or with steeper BMI trajectories during adulthood. All analyses were conducted separately for men and women as not only their weight, but also their BMI trajectories differed substantially (Table 1). We regarded correlations 0–0.39 as weak, 0.40–0.59 as moderate and 0.60–1.00 as strong. These analyses were performed using the R software (version 4.2.3).

**Table 1:** Means and standard deviations of BMI at different waves and BMI trajectories from waves 3 to 5 by sex.

	Men (*N* = 566)		Women (*N* = 834)		*P* value of sex difference
	Mean	SD	Mean	SD	
**Wave 1**					
Age (years)	11.42	0.30	11.42	0.29	0.70
BMI (kg/m^2^)	17.71	2.55	17.54	2.58	0.22
**Wave 2**					
Age (years)	14.05	0.08	14.04	0.08	<0.01
BMI (kg/m^2^)	19.34	2.67	19.35	2.63	0.39
**Wave 3**					
Age (years)	17.62	0.24	17.62	0.27	0.57
BMI (kg/m^2^)	21.77	2.94	20.93	2.72	<0.01
**Wave 4**					
Age (years)	24.18	1.65	24.27	1.63	0.06
BMI (kg/m^2^)	24.23	3.27	22.70	3.73	<0.01
**Wave 5**					
Age (years)	37.13	1.45	37.21	1.48	0.48
BMI (kg/m^2^)	26.16	3.68	25.02	4.36	<0.01
**BMI change**					
Slope (kg/m^2^ per year)	0.24	0.07	0.22	0.12	<0.01
Intercept (kg/m^2^)	21.77	2.80	20.99	2.74	<0.01

The slope BMI is the one obtained by the model based on BMI at 17.5, 24 and 37 years old, the intercept being the BMI estimated by the model at 17.5 years old.

*SD* standard deviation.

For assessing the underlying genetic and environmental contributions to the associations between BMI at different ages and its changes during adulthood, structural equation modelling was performed [27]. The methodology of classic twin modelling is based on the comparison between monozygotic twins (MZ) sharing 100% of their genetic variance (i.e., have virtually the same genomic sequence) and dizygotic (DZ) twins sharing, on average, 50% of their genetic variance (such as ordinary siblings) [28]. In accordance with these principles, the total variance of a trait can be decomposed into distinct components. The additive genetic component (A) encompasses all loci that influence the trait (A; correlation of 1 within MZ and 0.5 within DZ pairs). The dominance genetic component (D) is estimated if the DZ twin intra-class correlation (rDZ) is less than half of that of the MZ twin (rMZ). The shared or common environmental component (C) includes the entirety of the shared environment between co-twins that affects the trait (C; correlation 1 within both MZ and DZ pairs) and can be estimated along with the A component if the rDZ is greater than half of rMZ. Finally, the unique environmental component (E) includes those environmental factors that lead to the dissimilarity between co-twins and the measurement error (E; correlation 0 within both MZ and DZ twins).

Having only data from twins reared together, D and C components cannot be estimated simultaneously, and consequently only ACE, ADE, and their sub-models are available. To gain an initial understanding of the best model, we calculated intra-class correlations for DZ and MZ co-twins by dividing within-pair variation by between-pair variation estimated using analyses of variance. Supplementary Table 1 displays these within-pair correlations for BMI values at different ages and BMI changes by sex and zygosity. From this table, we infer that the most appropriate model is the ACE model. Finally, the ACE model can be compared in a subsequent stage to the simplified AE model. For all of these BMI variables except BMI at 37 years of age in both sexes, the correlations suggested an AE model. For BMI at 37 years of age common environmental or dominance genetic effects could also be present.

Genetic twin modelling assumes equal mean and variance for the first and second-born co-twin within a pair. These assumptions were evaluated by comparing the full ACE model to the saturated model which freely estimates all statistics without making any assumptions (Supplementary Table 2). Model fit was tested using -2 log likelihood (-2LL) statistics and comparing the difference of -2LL between models according to the difference between degrees of freedom. We found violations for BMI at age 17, intercept of BMI and slope of BMI (*p* < 0.0001) whereas for BMI at other ages, the genetic twin model fit well with the data. We further studied in detail by calculating means and SDs by sex and zygosity (Supplementary Table 3). However, we did not find any systematic differences in means or SDs between zygosity categories and generally, the differences were modest. Next, we compared different genetic models to find the best-fitting model. Three distinct submodels were constructed: (i) full models, (ii) models without sex-specific differences, and (iii) models without sex-specific genetic effects. Once the model was selected, the heritability (h^2^ = Va/V) and the proportion of total variance explained by shared and unique environmental components were calculated (Vc/V and Ve/V). The model fit statistics results indicated that the AE model with different parameters for men and women was the optimal model for slope, intercept, BMI at age 24 and BMI at age 37 (Supplementary Table 2). Conversely, the AE model with no sex differences was the selected model for BMI at age 11.5 and BMI at age 14. This was consistent with the descriptive information of our sample showing that significant differences in BMI emerged after the second wave (>14 years old) (Table 1).

Finally, a full Cholesky decomposition with four variables (BMI at age 12, BMI at age 14, intercept, and slope) was employed to analyse the covariation between these traits. Cholesky decomposition is a model-free method that decomposes all observed variation and covariation into uncorrelated latent factors. This method was used to decompose the covariances between the measures at different ages and the BMI trajectories into genetic and environmental covariances. Upon standardisation of the covariances, estimates of additive genetic (*r*_A_), shared environmental (*r*_C_) and unique environmental (*r*_E_) correlations can be obtained [29]. 95% confidence intervals (CI) were calculated using maximum likelihood estimation [29]. Genetic twin modelling was performed using the R software (version 4.2.3) and the R package OpenMx (version 2.21.11).

## Results

The mean BMI increased over time in both men and women, but the rates differed between sexes in adolescence (from 11.5 to 17.5 years of age) (4.06 kg/m^2^ in men and 3.39 kg/m^2^ in women) and in adults (from 17.5 to 35 years of age) (4.39 kg/m^2^ and 4.09 kg/m^2^, respectively) (Table 1). The sharpest increase occurred between ages 17.5 and 24 for men (2.46 kg/m^2^) and between ages 24 and 35 for women (2.32 kg/m^2^). Men exhibited a higher average BMI at most ages, but statistically significant sex differences emerged at the age of 17.5 and became more pronounced from the age of 24 to 37. In men, both the BMI growth velocity (i.e. slope) from 17.5 to 35 years of age (0.24 kg/m^2^ per year) and BMI at 17.5 years of age (21.77 kg/m^2^) were higher (*p* < 0.01) than in women (0.22 kg/m^2^ per year and 20.99 kg/m^2^, respectively).

Table 2 shows the heritability estimates for BMI at all ages as well as for BMI changes from 17.5 to 37 years of age (i.e. slope). The heritability of BMI was highest at 17.5 years of age for boys (h^2^ = 0.85) and at 12 years of age for girls (h^2^ = 0.84). Thereafter, it decreased systematically to 37 years of age (h^2^ = 0.72 in men and 0.75 in women). The proportion of BMI variation explained by unique environmental factors increased with age. Regarding the slope, the heritability estimate was found to be lower than that observed for distinct BMI values (h^2^ = 0.63 for men and 0.64 for women). The remainder of the variation was explained by unique environmental factors.

**Table 2:** Relative proportions of BMI variance explained by additive genetic and unique environmental variance components with 95% confidence intervals of BMI values and BMI trajectories in adulthood (from 17.5 to 37 years of age) by sex.

	Men						Women					
	Additive genetic component			Unique environmental component			Additive genetic component			Unique environmental component		
	a^2^	95% CI		e^2^	95% CI		a^2^	95% CI		e^2^	95% CI	
		LL	UL		LL	UL		LL	UL		LL	UL
BMI at 11.5	0.84	0.82	0.85	0.16	0.14	0.17	0.84	0.82	0.85	0.16	0.14	0.17
BMI at 14	0.84	0.81	0.85	0.16	0.14	0.18	0.84	0.81	0.85	0.16	0.14	0.18
BMI at 17.5	0.85	0.82	0.87	0.15	0.12	0.17	0.80	0.76	0.83	0.20	0.16	0.23
BMI at 24	0.75	0.69	0.79	0.25	0.20	0.30	0.80	0.77	0.83	0.20	0.16	0.22
BMI at 37	0.72	0.60	0.78	0.28	0.21	0.39	0.75	0.68	0.81	0.25	0.18	0.31
Slope	0.63	0.53	0.69	0.37	0.30	0.46	0.64	0.58	0.70	0.36	0.29	0.41
Intercept	0.81	0.77	0.86	0.19	0.14	0.25	0.78	0.73	0.82	0.22	0.17	0.23

AE model with sex differences was selected for the last three BMI values (17.5, 24 and 37 years old age), the Slope (the changes in BMI of these last three measures) and the intercept (BMI estimated by the model at 17.5 years old) while the AE model without sex differences was selected for the BMI values at the first two BMI values (11.5 and 14 years old).

*a*^*2*^ Heritability, *e*^*2*^ proportion of total variance explained by unique environmental factors, *LL* lower limit, *UL* upper limit.

Table 3 presents the phenotypic, additive genetic and environmental correlations between BMI values in adolescence (at age 11.5 and 14) and BMI trajectories (BMI at 17.5 years of age (intercept) and BMI change from 17.5 to 37 years of age (slope)). In both men and women, BMI at different ages (11.5, 14 and 17.5) showed weak correlations with BMI change from 17.5 to 37 years of age (*r* = 0.24–0.39). The observed phenotypic correlations were largely attributable to genetic factors, as evidenced by the higher additive genetic correlations (*r*_A_ = 0.35–0.85) compared to the phenotypic correlations. Conversely, the significant unique environmental correlations were relatively weak (0.15–0.39).

**Table 3:** Phenotypic, additive genetic and unique environmental correlations of BMI values during adolescence (ages 11.5 and 14 years old) and BMI trajectories during adulthood (from 17.5 to 37 years of age) by sex.

	Phenotypic correlation			Additive genetic correlation			Unique environmental correlation		
	*r*	95% CI		*r*_A_	95% CI		*r*_E_	95% CI	
		LL	UL		LL	UL		LL	UL
Men									
Slope vs intercept	0.24	0.16	0.32	0.50	0.42	0.60	−0.05	−0.18	0.09
BMI at 11.5 vs slope	0.26	0.18	0.34	0.42	0.34	0.49	0.16	0.03	0.28
BMI at 11.5 vs intercept	0.61	0.55	0.66	0.74	0.70	0.78	0.25	0.12	0.36
BMI at 14 vs slope	0.25	0.17	0.33	0.35	0.30	0.40	0.04	−0.10	0.17
BMI at 14 vs intercept	0.67	0.63	0.72	0.81	0.77	0.84	0.29	0.15	0.41
Women									
Slope vs intercept	0.35	0.29	0.41	0.54	0.51	0.58	0.07	−0.03	0.18
BMI at 11.5 vs slope	0.33	0.27	0.39	0.46	0.42	0.49	0.09	−0.01	0.20
BMI at 11.5 vs intercept	0.62	0.57	0.66	0.74	0.70	0.78	0.33	0.23	0.43
BMI at 14 vs slope	0.39	0.33	0.45	0.66	0.61	0.72	0.15	0.05	0.25
BMI at 14 vs intercept	0.70	0.67	0.74	0.85	0.82	0.87	0.39	0.29	0.48

Pairwise correlations between BMI measurements during adolescence and BMI trajectories during adulthood (from 17.5 to 37 years of age) which are defined as BMI changes (i.e. Slope) and BMI estimated by the model at 17.5 years old (i.e. Intercept) are summarised with correlation coefficients besides their confidence intervals.

*r* phenotypic correlation coefficient, *r*_*A*_ Additive genetic correlation coefficient, *r*_*E*_ Specific environmental correlation coefficient, *LL* Lower limit, *UL* Upper limit.

Finally, we studied the correlations between PRS_BMI_ and BMI at different ages (Table 4). PRS_BMI_ exhibited weak correlations with BMI at different ages (*r* = 0.24–0.31) as well as BMI trajectories (*r* = 0.25 in men and *r* = 0.27 in women). No systematic differences in these correlations were identified between men and women or between different age groups.

**Table 4:** Correlations between the PRS for BMI with BMI values and BMI changes during adulthood by sex.

	Men			Women		
	*r*	95% CI		*r*	95% CI	
		LL	UL		LL	UL
BMI at 11.5	0.24	0.17	0.31	0.28	0.22	0.35
BMI at 14	0.26	0.18	0.33	0.30	0.23	0.36
BMI at 17.5	0.28	0.21	0.36	0.24	0.17	0.30
BMI at 24	0.30	0.19	0.38	0.31	0.22	0.38
BMI at 37	0.29	0.18	0.39	0.29	0.19	0.39
Slope	0.25	0.13	0.35	0.27	0.17	0.36
Intercept	0.28	0.21	0.36	0.24	0.17	0.30

Pairwise correlations between BMI measurements, BMI trajectories during adulthood and the polygenic risk scores for the BMI are summarised with correlation coefficients besides their confidence intervals.

*PRS* polygenic risk score, *r* phenotypic correlation coefficient, *LL* lower limit, *UL* upper limit.

## Discussion

The current study provides insight into the relationship between BMI in adolescence and BMI changes during adulthood. Individuals with higher BMI at 11.5 and 14 years of age exhibited greater weight gain from 17.5 to 37 years of age. Furthermore, our findings indicate that these associations are primarily influenced by genetic factors, with some instances where environmental factors not shared by co-twins also play a role. Consequently, our study demonstrates the role of shared genetic factors in influencing both adolescent BMI and weight change in adulthood. Nevertheless, the increase in BMI during adulthood differed between sexes. The male participants exhibited a greater increase in BMI from the age of 17.5 to 37 years than the female participants, a finding that is consistent with the existing literature [30]. Heritability estimates of BMI during adolescence (from 11.5 to 14 years old) were 0.84 for boys and girls echoing previously reported heritability estimates from previous twin studies [31–33]. Heritability estimates of BMI during adulthood (from 17.5 to 37 years old) ranged from 0.72 to 0.85 in men and from 0.75 to 0.80 in women. These estimates are consistent with those reported in other studies conducted in the Finnish twin cohort [34] and in several European twin cohorts [29, 35]. This suggests that BMI during adolescence is more strongly influenced by genetic factors than BMI during adulthood [27].

Moderate phenotypic correlations were observed between BMI during adolescence (at 11.5 and 14 years of age) and BMI at 17.5 years of age. These results are in line with those of a previous study conducted in 2022, which used combined data from 25 longitudinal twin cohorts comprising 38,530 complete twin pairs and 283,766 longitudinal height and weight measures [29]. This suggests that BMI during adolescence (i.e. BMI at 11.5 and BMI at 14 years old) is related to BMI in early adulthood (i.e. BMI at 17.5 years old). Correlations between BMI at 11.5 years of age and BMI at 17.5 years of age were found to be influenced by both genetic and environmental factors in both men and women. Similarly, we observed genetic and environmental correlations between BMI at 14 years of age and BMI at 17.5 years of age in both men and women. A study carried out by Choh et al. [36] using 1176 participants from the USA [36] reported a high genetic correlation between BMI at 19 years of age and at 15 years of age (*r*_A_ = 0.84). Additionally, a previously mentioned study carried out in 2022 with data from 25 longitudinal twin cohorts (*N* = 38,530 twins and 283,766 longitudinal height and weight measures) found that not only phenotypic correlation between BMI at 11 and 17 years but also the one between BMI at 14 and 17 years old was driven in both sexes by both components, genetic (BMI11-BMI17: *r*_A_ = 0.75 in boys and 0.71 in girls. BMI14-BMI17: *r*_A_ = 0.84 in boys and 0.86 in girls) and environmental (BMI11-BMI17: *r*_E_ = 0.25 in boys and 0.40 in girls. BMI14-BMI17: *r*_E_ = 0.26 in boys and 0.29 in girls) [29]. These genetic and environmental correlations were consistent with those we report in this study between BMI at 14 years of age and BMI at 17.5 years of age.

Correlations were found between BMI measurements during adolescence and the subsequent changes in BMI during adulthood in both men and women. This evidence substantiates the assertion that BMI in adolescence is a contributing factor in the alteration of BMI values during adulthood. Regarding the phenotypic correlation between BMI at 11.5 years of age and the changes in BMI during adulthood, we showed that it was explained by both genetics and the environment in men, but in women, the environment did not play a significant role. The opposite was found for the phenotypic correlation between BMI at 14 years of age and changes in BMI during adulthood. In men, the genetic component was the sole contributor to the observed correlation, with the environmental factors playing a role but not reaching statistical significance. In women, both genetic and environmental factors were found to be statistically significant. The observation that all environmental correlations are positive, despite not being systematically significant, suggests that the lack of significance is likely due to insufficient statistical power. The observed correlations between BMI at age 17.5 (i.e. the intercept) and the changes in BMI during adulthood were explained only by genetics in both sexes, as the environmental component was not statistically significant. A previous study based on another Finnish twin cohort comprising 10,556 twin individuals aged 18 or more at baseline with self-reported weight and height from three-time points (1975, 1981 and 1990) found a weak additive genetic correlation between intercept and slope in men and women, with the correlation being negative and significant only in men [17]. Moreover, this previous study also reported a significant unique environmental correlation between intercept and slope in women, but not in men. These results are thus contrary to those found in our study, which might be explained by the differences in the data, the age of participants and the time when the data was collected.

Finally, the correlations between PRS_BMI_, BMI at different ages, and BMI changes over adulthood were found to be weak in both sexes. The strength of these correlations ranged from 0.24 to 0.30 in men and 0.24 to 0.31 in women. These results demonstrate that the genetic factors underlying BMI variation influence BMI and its changes. Similar results were previously obtained in other twin studies [37, 38]. Secondly, correlations between PRS_BMI_ and BMI in adolescence (ages 11.5 and 14), even though weak, suggest that some genetic factors underlying BMI are shared between adolescence and adulthood indicating that the genetic factors behind BMI variation in adolescence and adulthood partly overlap. Finally, these results open the door to studying whether the genetic basis of adult BMI is also linked to BMI and its fluctuations during childhood.

The current study has strengths but also limitations. The most important strength of this study is the use of longitudinal data with a follow-up from the pubertal stage to early midlife, spanning over two decades and five measurement points, to which genetic information depicting predisposition to obesity was added. However, as we sought to take advantage of the multiple measurements of BMI over time, we ensured that only individuals with BMI measurements at ages 17.5, 24, and 37 years old were included, which resulted in a sharp decrease in sample size. Consequently, this might have substantially decreased statistical power and increased uncertainty in our estimates. Moreover, the use of a relatively small number of BMI measurements does not allow for a more complex vision of how individuals gain weight. Some individuals are rather stable in weight and pattern of weight gain, while others have a great deal of weight cycling, albeit over an overall trajectory of weight gain. Another limitation is the use of linear models to estimate changes in BMI during adulthood, as BMI trajectories may exhibit nonlinear patterns. Thus, the assumption that changes in BMI are linear in time is a limitation. However, our study design does not allow for a proper investigation of nonlinearity in changes in BMI, as we had a modest sample size and used only 3 measurements of BMI per individual to fit slope values. The participants having genetic information had lower BMIs showing self-selection. However, the results based on PRS and classical twin modelling still showed the role of shared genetic factors behind BMI in adolescence and weight gain in adulthood. Finally, BMI although being a widely used measure in the field of obesity, does not provide information on the body composition of individuals as other measures do, such as body fat percentage or body adiposity index.

In conclusion, the current study provides evidence that BMI during adolescence and changes in BMI during adulthood are genetically correlated and that the environment explains only a small portion of the phenotypic associations. In addition, we demonstrated that a predisposition to obesity in early adolescence is a good predictor of BMI change in adulthood. This knowledge is important when identifying those who are at the highest risk for accumulating weight over adulthood.

## Supplementary information

Supplementary material

## Data Availability

The FinnTwin12 data is not publicly available due to the restrictions of informed consent. However, the FinnTwin12 data is available through the Institute for Molecular Medicine Finland (FIMM) Data Access Committee (DAC) (fimmdac@helsinki.fi) for authorised researchers who have IRB/ethics approval and an institutionally approved study plan. To ensure the protection of privacy and compliance with national data protection legislation, a data use/transfer agreement is needed, the content and specific clauses of which will depend on the nature of the requested data. The ethics committee of the Helsinki University Central Hospital District (HUS) approved the most recent data collection (wave 5) (HUS/2226/2021, dated September 22, 2021) and the use of prior collected data.

## Code availability

All computer codes are available from the corresponding author.
