# Supplementary material for: Genetic contributions to body mass index over adolescence and its associations with adult weight gain: a 25-year follow-up study of Finnish twins
Source: Int J Obes (Lond). 2024 Nov 20;49(2):357–63. doi: 10.1038/s41366-024-01684-3 (PMC11805703; doi:10.1038/s41366-024-01684-3)
Supplement: Supplementary file 1 — Supplementary material [file 41366_2024_1684_MOESM1_ESM.docx]

**Supplementary table 1:** Intraclass correlations for the BMI at the different surveys and trajectories during adulthood (from ages 17.5 to 37 years) by sex and zygosity.

|  | Men (N of pairs=173) | | | | | | Women (N of pairs=175) | | | | | | OSDZ  (N of pairs=146) | | |
| --- | --- | --- | --- | --- | --- | --- | --- | --- | --- | --- | --- | --- | --- | --- | --- |
|  | MZ (N of pairs=103) | | | DZ (N of pairs=70) | | | MZ(N of pairs=97) | | | DZ (N of pairs=78) | | |  |  |  |
|  | Correlation | 95%CI | | Correlation | 95%CI | | Correlation | 95%CI | | Correlation | 95%CI | | Correlation | 95%CI | |
|  |  | LL | UB |  | LL | UB |  | LL | UB |  | LL | UB |  | LL | UB |
| **BMI measures and changes** |  |  |  |  |  |  |  |  |  |  |  |  |  |  |  |
| BMI at 11.5 | 0.82 | 0.79 | 0.86 | 0.49 | 0.43 | 0.56 | 0.81 | 0.77 | 0.84 | 0.49 | 0.40 | 0.56 | 0.43 | 0.37 | 0.49 |
| BMI at 14 | 0.83 | 0.79 | 0.86 | 0.52 | 0.44 | 0.59 | 0.80 | 0.76 | 0.83 | 0.45 | 0.36 | 0.53 | 0.39 | 0.33 | 0.45 |
| BMI at 17.5 | 0.82 | 0.77 | 0.85 | 0.37 | 0.27 | 0.47 | 0.77 | 0.72 | 0.81 | 0.35 | 0.25 | 0.44 | 0.31 | 0.24 | 0.38 |
| BMI at 24 | 0.75 | 0.68 | 0.80 | 0.36 | 0.22 | 0.48 | 0.79 | 0.75 | 0.83 | 0.40 | 0.29 | 0.50 | 0.29 | 0.19 | 0.38 |
| BMI at 37 | 0.66 | 0.52 | 0.77 | 0.46 | 0.22 | 0.65 | 0.75 | 0.66 | 0.82 | 0.48 | 0.29 | 0.63 | 0.26 | 0.09 | 0.41 |
| Slope BMI | 0.58 | 0.48 | 0.66 | 0.33 | 0.19 | 0.46 | 0.66 | 0.59 | 0.72 | 0.31 | 0.20 | 0.42 | 0.22 | 0.12 | 0.31 |
| Intercept BMI | 0.76 | 0.70 | 0.81 | 0.46 | 0.33 | 0.57 | 0.78 | 0.72 | 0.81 | 0.41 | 0.30 | 0.50 | 0.22 | 0.12 | 0.31 |

**Caption**: Intraclass correlations for BMI measurements, the BMI baseline (BMI estimated by the model at 17.5 years old) and the changes in BMI during adulthood (from ages 17.5 to 37 years) are summarized by sex and zygosity with correlation coefficients besides their 95% confidence interval. All the correlations are highly significant (all p<0.005). **Abbreviations:** MZ: Monozygotic; DZ: Dizygotic; OSDZ: Opposite sex dizygotic. LL: Lower limit; UL: Upper limit; BMI: Body mass index.

**Supplementary table 2:** Model fit statistics of BMI measures and trajectories and underlying factors comparing different genetic models.

|  | Saturated model  (reference model) | | Full ACE model (1) | | ACE model without sex-specific genetic effect (2) | | ACE model with same parameter estimates for boys and girls (3) | | Full AE model (4) | | AE model without sex-specific genetic effect (5) | | AE model with same parameter estimates for boys and girls (6) | |
| --- | --- | --- | --- | --- | --- | --- | --- | --- | --- | --- | --- | --- | --- | --- |
|  | 2LL | d.f | Δ -2 LL | p value | Δ -2 LL | p value | Δ -2 LL | p value | Δ -2 LL | p value | Δ -2 LL | p value | Δ -2 LL | p value |
| **BMI measures and changes** |  |  |  |  |  |  |  |  |  |  |  |  |  |  |
| BMI at 11.5 | 20527.04 | 4583 | 21.94 | 0.14 | 2.18 | 0.13 | 2.29 | 0.51 | 6.25 | 0.04 | 0.04 | 0.82 | 2.32 | 0.12 |
| BMI at 14 | 19382.44 | 4241 | 19.65 | 0.23 | 2.84 | 0.09 | 3.81 | 0.28 | 6.98 | <0.01 | 1.87 | 0.17 | 2.19 | 0.13 |
| BMI at 17.5 | 18537.67 | 3914 | 45.49 | 1.17e-04 | 4.00 | 0.04 | 14.59 | <0.01 | <0.01 | 1.00 | 10.88 | <0.01 | 3.70 | 0.05 |
| BMI at 24 | 16074.54 | 3080 | 22.28 | 0.13 | 0.03 | 0.85 | 23.29 | <0.01 | 0.47 | 0.78 | 7.87 | <0.01 | 15.38 | <0.01 |
| BMI at 37 | 9957.15 | 1774 | 10.133 | 0.85 | 1.95 | 0.16 | 22.36 | <0.01 | 2.46 | 0.29 | 64.30 | <0.01 | -42.23 | 1.00 |
| Slope BMI | 22709.28 | 3115 | 49.51 | 2.74e-05 | 0.03 | 0.86 | 310.25 | <0.01 | 0.33 | 0.84 | 4.91 | 0.02 | 305.27 | <0.01 |
| Intercept BMI | 22741.78 | 3115 | 49.59 | 2.66e-05 | 2.80 | 0.10 | 12.6 | <0.01 | 1.34 | 0.63 | 1.34 | 0.26 | 4.90 | 0.02 |

**Caption:** Full ACE model is compared against the rest of the models displaying the -2 log likelihood and degrees of freedom for the reference model and the differences in -2log likelihood and the p value in the models compared with the reference one. **Abbreviations:** -2LL (-2 log-likelihood); d.f. (degrees of freedom); Δ (change); ACE (additive genetic/ shared environment/ unique environment) model; AE (additive genetic/ unique environment) model.(1) Compared to saturated model (Δ d.f. 16); (2) Compared to full ACE model (Δ d.f. 1); (3) Compared to ACE model without sex-specific genetic effect (Δ d.f. 2); (4) Compared to full ACE model (Δ d.f. 2); (5) Compared to the full AE model (Δ d.f. 1); (6) Compared to the AE model without sex-specific genetic effect (Δ d.f. 1); BMI: Body mass index.

Supplementary table 3: Means and Standard deviations of BMI at the different surveys and its changes during adulthood (from ages 17.5 to 37 years) by sex and zygosity.

|  | Men | | | | | | Women | | | | | |
| --- | --- | --- | --- | --- | --- | --- | --- | --- | --- | --- | --- | --- |
|  | MZ | | SSDZ | | OSDZ | | MZ | | SSDZ | | OSDZ | |
|  | Mean | SD | Mean | SD | Mean | SD | Mean | SD | Mean | SD | Mean | SD |
| **BMI measures and changes** |  |  |  |  |  |  |  |  |  |  |  |  |
| BMI at 11.5 | 17.59 | 2.43 | 17.82 | 2.59 | 17.79 | 2.63 | 17.42 | 2.46 | 17.58 | 2.58 | 17.68 | 2.70 |
| BMI at 14 | 19.16 | 2.54 | 19.51 | 2.81 | 19.34 | 2.72 | 19.22 | 2.59 | 19.33 | 2.55 | 19.51 | 2.75 |
| BMI at 17.5 | 21.46 | 2.65 | 22.08 | 3.05 | 21.84 | 3.13 | 20.70 | 2.62 | 20.97 | 2.74 | 21.13 | 2.81 |
| BMI at 24 | 24.06 | 3.28 | 24.41 | 3.32 | 24.37 | 3.27 | 22.48 | 3.62 | 22.74 | 3.60 | 22.95 | 4.00 |
| BMI at 37 | 26.06 | 3.57 | 26.57 | 3.80 | 25.82 | 3.69 | 24.81 | 4.32 | 25.32 | 4.40 | 25.05 | 4.42 |
| Slope BMI | 0.24 | 0.070 | 0.25 | 0.077 | 0.24 | 0.072 | 0.21 | 0.117 | 0.22 | 0.111 | 0.22 | 0.122 |
| Intercept BMI | 21.48 | 2.49 | 21.97 | 2.84 | 21.89 | 3.07 | 20.78 | 2.59 | 21.07 | 2.86 | 21.16 | 2.82 |

**Caption**: Mean and standard deviation of body mass index and its changes from the individuals included in the study aresummarized by sex and zygosity. **Abbreviations:** BMI: Body mass index; SD: Standard deviation; MZ: Monozygotic; DZ: Dizygotic; SSDZ: Same-sex dizygotic; OSDZ: Opposite sex dizygotic.
